# Climatic and societal impacts of a “forgotten” cluster of volcanic eruptions in 1108-1110 CE

**DOI:** 10.1038/s41598-020-63339-3

**Published:** 2020-04-21

**Authors:** Sébastien Guillet, Christophe Corona, Francis Ludlow, Clive Oppenheimer, Markus Stoffel

**Affiliations:** 1 Climatic Change Impacts and Risks in the Anthropocene (C-CIA), Institute for Environmental Sciences, University of Geneva, 66 Boulevard Carl Vogt, CH-1205 Geneva, Switzerland; 2 Geolab, Université Clermont Auvergne, CNRS, F-63000 Clermont-Ferrand, France; 3 Trinity Centre for Environmental Humanities, Department of History, School of Histories & Humanities, Trinity College, Dublin, D2 Ireland; 4 Department of Geography, University of Cambridge, Downing Place, Cambridge, CB2 3EN United Kingdom; 5 Department of Earth Sciences, University of Geneva, Rue des Maraîchers 13, CH-1205 Geneva, Switzerland; 6 Department F.-A. Forel for Environmental and Aquatic Sciences, University of Geneva, 66 Boulevard Carl Vogt, CH-1205 Geneva, Switzerland

**Keywords:** Cryospheric science, Palaeoclimate

## Abstract

Recently revised ice core chronologies for Greenland have newly identified one of the largest sulfate deposition signals of the last millennium as occurring between 1108 and 1113 CE. Long considered the product of the 1104 CE Hekla (Iceland) eruption, this event can now be associated with substantial deposition seen in Antarctica under a similarly revised chronology. This newly recognized bipolar deposition episode has consequently been deemed to reveal a previously unknown major tropical eruption in 1108 CE. Here we show that a unique medieval observation of a “dark” total lunar eclipse attests to a dust veil over Europe in May 1110 CE, corroborating the revised ice-core chronologies. Furthermore, careful evaluation of ice core records points to the occurrence of several closely spaced volcanic eruptions between 1108 and 1110 CE. The sources of these eruptions remain unknown, but we propose that Mt. Asama, whose largest Holocene eruption occurred in August 1108 CE and is credibly documented by a contemporary Japanese observer, is a plausible contributor to the elevated sulfate in Greenland. Dendroclimatology and historical documentation both attest, moreover, to severe climatic anomalies following the proposed eruptions, likely providing the environmental preconditions for subsistence crises experienced in Western Europe between 1109 and 1111 CE.

## Introduction

While understanding of the climate forcing associated with large volcanic eruptions has advanced greatly since the 1991 Mount Pinatubo eruption, the wider environmental impacts and consequences of volcanism on the trajectories of human societies remain only partially explored, especially for the early and high medieval periods (*c*. 400–1300 CE). Historical investigations have been mostly restricted to a limited number of volcanic events such as the 939 Eldgjá, 946 Changbaishan and 1257 Samalas eruptions^1–5^. The paucity of studies covering the high medieval period can be explained by (i) the challenges in accessing, reading, and interpreting European, Near Eastern and Asian historical sources, (ii) the limited number of well-replicated millennium-length tree-ring chronologies, and (iii) uncertainties in ice core dating.

Using high-resolution ice core glacio-chemical measurements, automated ice-core layer counting, well-dated and independent time markers (including the cosmic-ray events of 774 and 993 CE), tephra markers and historical reports of atmospheric phenomena (e.g., dust veils) associated with volcanic aerosol presence, Sigl *et al*.^6^ demonstrated that ice core records relying upon the Greenland Ice Core Chronology 2005 (GICC05) timescale required a correction of approximately seven years in the first millennium CE, and up to four years in the period we study in this paper. This led to revised ice-core chronologies for Greenland (NS1–2011) and Antarctica (WD2014), as well as to refinements of the radiative forcing record (due to volcanism) extending back to 500 BCE.

A prominent discovery arising from this revised ice-core dating is a major and hitherto unrecognized bipolar volcanic signal with sulfate deposition starting in late 1108 or early 1109 CE and persisting until early 1113 CE in the Greenland record^6^. Using the GICC05 timescale, this volcanic horizon in Greenland had previously been ascribed to the 1104 CE Hekla eruption^6–9^ – although no tephra has yet been identified in this layer in any Greenland ice core^6,9,10^ – and thereby disassociated from what had appeared to be a slightly younger sulfate peak in Antarctic ice core records. The same signal in the Greenland Ice Sheet Project 2 (GISP2) ice core^11^ was similarly ascribed to Hekla at 1104 CE and used as a chronological tie-point for the Meese-Sowers GISP2 time-scale. Examining whether the re-dating of this volcanic horizon is supported by independent evidence is therefore critical when assessing the validity of ice-core timescales (e.g., Meese-Sowers, GICC05, NS1–2011). With an estimated volcanic stratospheric sulfur injection (VSSI) of 19.2 Tg[S] and a cumulative global radiative forcing of −12.6 W/m^2^, the event ranks seventh in the last millennium or 13^th^ in the last 2500 years in the NS1–2011 volcanic forcing dataset^6,12^. This degree of forcing is comparable with that estimated for the major volcanic signal in *c*. 1601 CE (−11.6 W/m^2^), for which there is evidence for significant impacts on climate and society^13–16^. Here using historical and tree ring records we investigate whether independent evidence exists both for the existence and timing of the 1108 CE event as proposed in the NS1–2011 timescale. We also evaluate the potential eruptive source(s) and the impacts that this event may have had on NH climate and contemporary societies.

### Dark total lunar eclipse corroborates timing of major explosive volcanism

The spectacular atmospheric optical phenomena associated with high-altitude volcanic aerosols have caught the attention of chroniclers since ancient times. Relevant observations include twilight glows, dimming or discoloration of the Sun, and solar coronae (i.e., Bishop’s Rings)^17,18^. Reports of these manifestations have been used to identify the occurrence, timing, and climatic-forcing potential of large eruptions^19,20^, and to validate and anchor ice core chronologies^6^. Volcanic influences on the appearance of the lunar disk hold similar potential, though have received less attention. In particular, the reported brightness of lunar eclipses can be employed both to detect volcanic aerosols in the stratosphere and to quantify stratospheric optical depths following large eruptions^4,21–24^.

The darkest total lunar eclipses (Luminosity = 0, see Materials and Methods) recorded since 1600 CE (i.e. in 1601, 1642, 1816, 1884, 1913, 1983, and 1992) have all been linked to large volcanic eruptions, namely the 1600 Huaynaputina, 1641 Parker, 1815 Tambora, 1883 Krakatau, 1912 Katmai-Novarupta, 1983 El Chichón, and 1991 Pinatubo eruptions^21,23,24^. Despite this potential, high medieval written sources have yet to be fully examined for contemporary evidence of dark total lunar eclipses. Accordingly, we thoroughly surveyed available European and Near Eastern texts spanning the early 12^th^ century. NASA’s *Five Millennia Catalog of Lunar Eclipses*^25^ (based on astronomical retrocalculation) notes that seven total lunar eclipses were observable in Europe between 1100 and 1120 CE, namely on 17 September 1103, 11 January 1107, 6 July 1107, 5 May 1110, 8 August 1114, 16 June 1117 and 10 December 1117 CE. Our survey recovered 17 original historical reports that credibly pertain to these eclipses.

In total, six out of the 17 observations specified the color and brightness of the Moon and allowed characterization of eclipses according to the Danjon scale, which ranks lunar luminosity on a scale of 0 to 4 in order of increasing brightness (see Materials and Methods); all eclipses - with documented brightness - were rated bright at L = 3 or L = 4 (Table S1), except that of 1110 CE, for which L = 0.

The contemporary Anglo Saxon *Peterborough Chronicle* highlights the exceptional darkness of the Moon in this latter case^26^: “*On the fifth night in the month of May appeared the moon shining bright in the evening, and afterwards by little and little its light diminished, so that, as soon as night came, it was so completely extinguished withal, that neither light, nor orb, nor anything at all of it was seen. And so it continued nearly until day, and then appeared shining full and bright*.” Significantly, the chronicler attests to an otherwise clear night sky, indicating that the Moon’s disappearance cannot be explained by the presence of clouds: “*All the night was the firmament very clear, and the stars over all the heavens shining very bright.”* This testimony contrasts starkly with records of blood-red Moons for previous and subsequent eclipses (Table S1). We also note that this is one of the longest and most detailed accounts we are aware of for any dark lunar eclipse occurring between 500 and 1800 CE, rivalling the apparent darkness of the total lunar eclipses noticed after the 1257 CE Samalas^5^ and 1883 CE Krakatau^27–29^ eruptions (see Text S1 for more details).

The darkness of the 1110 CE total lunar eclipse has, indeed, long caught the attention of astronomers^21,30–32^. The astronomer Georges Frederick Chambers (1841–1915) was one of the first to remark on its singular nature: “*It is evident that this [eclipse] was an instance of a* *‘black’ eclipse when the Moon becomes quite invisible instead of shining with the familiar coppery hue*”^30^. Hitherto, the blackness of this eclipsed Moon has not been directly linked to the presence of volcanic aerosols in the stratosphere in May 1110 CE. The new evidence for a stratospheric aerosol veil in 1110 CE thus provides an independent validation of the NS1–2011 and WD2014 timescales, which place notable sulfate deposition in both poles at this time. We note that no other evidence of volcanic dust veil, such as a dimming of the Sun, red twilight glows and/or reddish solar haloes, could be found during our investigations for the years 1108–1110 CE.

### Reinterpreting the bipolar ice core signature and evaluating potential eruptive source(s)

Sigl. *et al*.^6^ originally assigned the 1108–1109 CE volcanic ice core horizon to one single unidentified tropical eruption. Here, based on a close re-examination of the NEEM-2011-S1, NGRIP and WDC06A records, we posit that this volcanic horizon may instead reflect the contribution of several closely-spaced volcanic eruptions that occurred between 1108 and 1110 CE.

**Figure 1:**
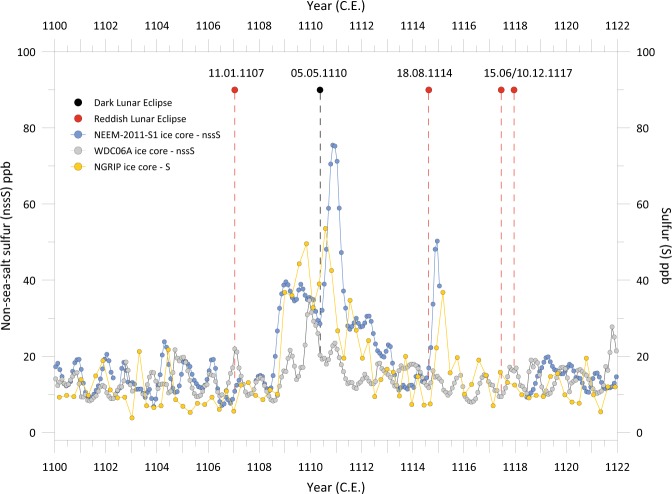
(a) Monthly-resolved non-sea-salt sulfur records from the NEEM-2011-S1, NGRIP and the WDC06A ice cores^6^ and (b) brightness of the total lunar eclipses observed in Europe for the period 1100–1122 CE. Dark total eclipses of the Moon indicate high atmospheric turbidity; while a reddish disk shown by the eclipsed Moon betrays a clear stratosphere.

The NEEM-2011-S1 record shows a sharp inflection upward around mid-1108 CE, reaching a peak (~40 nssS) by the end of the year (Fig. 1). The signal then gradually decays until around mid-1110 CE when there is another substantial increase in sulfur to a peak (~75 nssS) in late 1110 CE followed by a sharp decline to early 1111 CE. Lesser but still elevated sulphate then persists until late 1112 or early 1113 CE. The NGRIP record – synchronized to the NS1–2011 timescale (Sigl *et al*., 2015) – largely follows, despite its lower resolution and some short-lived departures, the NEEM-2011-S1 trend (Fig. 1). The signal in the WDC06A record is less pronounced than in the Greenland ice cores. It shows elevated sulfur deposition most visibly between 1109 and 1111 CE, with a main peak (~35 nssS) in *c*. late 1109 CE (Fig. 1). Allowing for uncertainty in the inter-polar chronologies, it is possible to reach several credible interpretations of the volcanic history over these years, summarized as scenarios 1–3 in the following:

**Scenario 1:** (1) a tropical eruption in 1108 CE, responsible for the sulfur deposits in Antarctica in 1109 CE and contributing to the broader signal (1108–1113 CE) seen in the Greenland records, together with (2) an almost synchronous high latitude NH eruption (potentially of Asama in Japan in August-October 1108 CE, see below for details), and (3) a further high-latitude NH eruption in late 1109 or early 1110 CE.

**Scenario 2:** (1) A number of large eruptions in the mid- to high latitudes of the NH (Mount Asama being one potential source), with deposition starting in 1108 CE, responsible for the broader signal (1108–1113 CE) seen in Greenland, and (2) a tropical eruption with sulfur deposition peaking in late 1109 CE in the WDC06A ice core and contributing additional volcanic aerosol deposition in Greenland records.

**Scenario 3:** Alternatively, one could also propose a scenario without any tropical eruption. This scenario would include: (1) A number of large eruptions in the mid- to high latitudes of the NH (Mount Asama being one potential source, as before), with deposition starting in 1108 CE, responsible for the extended period of sulfur deposition (1108–1113 CE) seen in the Greenland records. (2) A large eruption in the Southern Hemisphere (SH) in late 1108 to early 1109 CE, independently producing the elevated levels of sulfur deposition observed in the WDC06A ice core.

At this stage, the temporal resolution of the NEEM-2011-S1 and WDC06A ice core records does not allow more precise estimation of the number of volcanic eruptions that contributed to sulfur deposition between 1108 and 1113 CE, nor does it allow for the quantification of the individual contribution of each eruption to this deposition. The sources of these eruptions also remain unknown, yet one eruption with a historical date in this period is that of Mount Asama in Japan (36°40′ N, 138°52′ E, 2568 m above sea level). The so-called *Chūyūki* 中右記 diary, written by the statesman Fujiwara no Munetada 藤原宗忠 (1062–1141), states that the eruption of Mount Asama began in late August 1108 and lasted until October 1108 CE: *“October 13: According to a report from the province of Kōzuke, there is a high mountain in the middle of the province, Mount Asama. In the years 1065–1069, a slight smoke rose above the volcano but later became imperceptible. On August 29, there was a fire at the top of the volcano, a thick layer of ash in the governor’s garden, everywhere the fields and the rice fields are rendered unfit for cultivation. We never saw that in the country. It is a very strange and rare thing.*” (see Table S2 for more details). With an assigned VEI of 5, the 1108 CE eruption is considered the largest Holocene eruption of Asama. Its magnitude is larger than that of the volcano’s notorious 1783 CE eruption, which killed more than 1400 people. Taking the eruption period as August-October of 1108 CE, associated sulfate deposition over Greenland can be expected to have potentially spanned from late 1108 CE up to early 1110 CE. Considering chronological uncertainties, we consider it plausible that the 1108 CE Asama eruption contributed to the Greenland ice core signal. The occurrence of several major volcanic events with a NH ice-core sulphate deposition bias is also consistent with a heavy NH stratospheric aerosol loading sufficient to induce the May 1110 CE dark eclipse.

### Tree-ring-based evidence of climatic impacts of the 1108–1110 CE volcanic cluster

To identify as well as spatially and temporally characterize potential climatic impacts arising from these eruptions, which we describe as the 1108-1110 CE volcanic cluster, we employ a tree-ring network of 25 NH chronologies (Table S3, Fig. S1^5^). These are used to reconstruct JJA temperatures for 40–90°N over land and spanning the period 500–2000 CE (see Table S4 for statistical details of the reconstruction).

**Figure 2:**
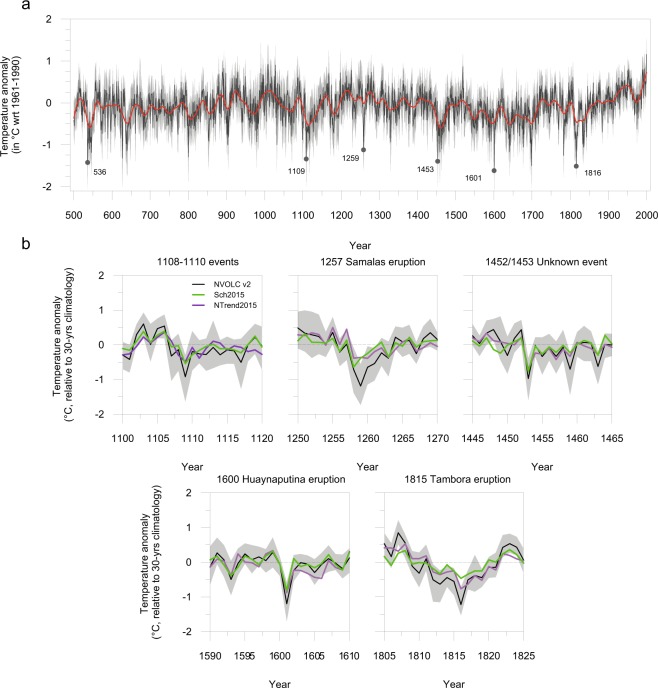
Tree-ring reconstructions *(NVOLC v2)* of NH extra-tropical land (40–90°N) summer temperature anomalies (with respect to the period 1961–1990) since 500 CE. (**a**) Comparison of *NVOLC_filt v2* with recently published reconstructions, *Sch2015* (^33^) and (**b**) *N-TREND2015* (^34^) for the 1108–1110 CE, 1257 CE Samalas, 1452/1453 CE Unknown, 1600 CE Huaynaputina, and 1815 CE Tambora eruptions.

**Figure 3:**
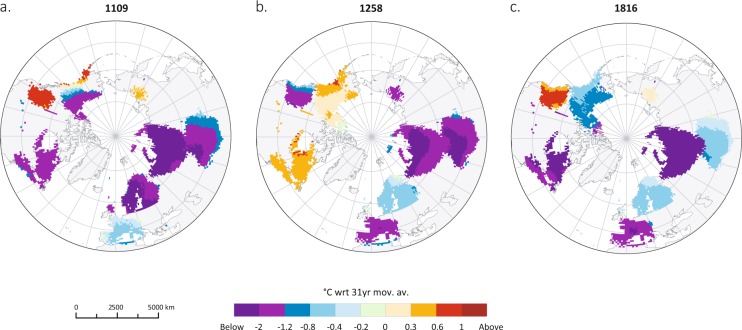
Spatial extent of the JJA temperature anomalies induced by (**a**) the 1108–1110 CE, (**b**) 1257 CE Samalas and (**c**) 1815 CE Tambora eruptions.

The unfiltered reconstruction (Fig. 2a; Material and Methods) identified 1109 CE as experiencing one of the most extreme NH summer coolings of the last ~1500 years (rank 6) with temperatures of −1.3 °C relative to the 1961–1990 reference period. This value is exceeded by only a handful of cases, all of which are associated with major explosive volcanism, including −1.6 °C in 1601 CE (rank 1, Huaynaputina), −1.5 °C in 1816 CE (rank 3, Tambora), −1.4 °C in 536 CE (rank 4, unidentified volcano) and –1.3 °C in 1453 CE (rank 5, unidentified volcano). Examining the filtered *NVOLC_filt* *v2* reconstruction, designed to quantify volcanically-induced cooling with respect to contemporary climatology (see Material and Methods), strong NH summer cooling is still seen to prevail in 1109 CE (−1 °C; Fig. 2b), being the 11^th^ coldest NH summer since 500 CE. Comparable values are observed for the years 1816, 1601, 1259 (all at −1.2 °C), 536 (−1 °C), and 1453 CE (−0.9 °C), all associated with major volcanic eruptions (Fig. 3b), and ranking as the 1^st^, 3^rd^, 2^nd^, 7^th^, and 12^th^ coldest years in the filtered series, respectively. Our findings are corroborated by comparison with other recent, large-scale reconstructions^33,34^ (hereafter referred to as *Sch015* and *NTREND015*) that rank 1109 CE as the 12^th^ and 30^th^ coolest summer, respectively, of the last millennium (Fig. 2b). Notably, none of these reconstructions (*NVOLC v2*, *NVOLC_filt v2*, *Sch015* and *NTREND015*) record significant summer temperature anomalies at the hemispheric scale in the years 1110 or 1111 CE (Fig. 2b).

Figures 3 and S2 show JJA-gridded reconstructions for 1109 CE (3065 1°×1° grid points with a coefficient of efficiency>0.1) that reveal widespread, but heterogeneous NH summer cooling. The most affected regions are Siberia (−2.3 °C), Scandinavia (−2 °C), Central Asia (−1.5 °C), Quebec (−1.5 °C) and, to a lesser extent, Western Europe (−0.4 °C). This evidence for particularly severe cooling in Siberia, Central Asia, and Quebec is corroborated by the occurrence of frost rings and light rings in the tree-ring chronologies of Yamal, Polar Ural, Altai, Quebec and Western USA during the 1109 CE growing season (i.e. April-September; Table S5). In contrast, above-average temperatures prevailed in British Columbia (+0.8 °C), Greenland (+0.4 °C), and Yakutia (+0.3 °C) during this summer (Fig. 3). The persistence of the summer cooling following our proposed eruption cluster between 1108 and 1110 CE is also heterogeneous in spatial terms. Whereas Siberia, Scandinavia, and Central Asia experienced the most extended cooling (>2 years), elsewhere reconstructed summer temperatures recovered more rapidly after 1109 CE (Fig. S3).

### Documentary evidence for climatic impacts in Western Europe

We identified 13 narrative sources (Table S2) that describe meteorological phenomena and conditions in the years 1109–1111 CE. Despite the more limited frequency of meteorological entries at the beginning of the 12^th^ century when compared with subsequent periods^35,36^, the abundance of testimonies referring to adverse weather, crop failures, and famines in these years is notably comparable with the number of accounts for later events such as 1137 CE, known as one of the driest summers of the century, which had major human impacts, or with 1151, 1174, 1195, and 1196 CE (see Fig. S4), which stand out as the coldest and wettest summers of this period^37,38^ (Fig. S5).

The majority of our surveyed sources describe heavy and unseasonable rainfalls over the Atlantic domain of Europe, especially in the summer and autumn of 1109 CE. For instance, testimony from France provided by Orderic Vitalis (1075–1141/43 CE), a monk at the Saint-Evroult abbey and a contemporary witness, describes “*excessive rainfall”*, while the *Ex Historiae Francicae Fragmento* and the *Annals of Hasungen* similarly report continuous rainfall in the North and Western part of France and in Germany (Table S2). For Ireland, the contemporary Gaelic Irish *Annals of Inisfallen* report *“heavy rain and bad weather in the summer and autumn”* (Table S2). The hydroclimatic anomalies experienced during the summer of 1109 CE are for this region of Europe consistent with climate observations in 1258 and 1816 CE after the Samalas and Tambora eruptions, respectively^4,5,39–42^. The Old World Drought Atlas (OWDA)^43^, which employs tree-ring data to reconstruct spring-summer soil moisture, also corroborates our documentary evidence for wet summer conditions in Ireland, Britain, and Western France for 1109 CE (Fig. S6). The overt focus of our documentary data on precipitation anomalies (through their impacts on flooding and crop failures), as opposed to summer temperature anomalies, is notable and usefully demonstrates how sources may exhibit biases towards reporting the meteorological phenomena with the greatest societal impact^44^. We note, however, that over Northwest Europe, years with wet spring-summer conditions are usually also cold^45^, such that our documentary evidence of anomalously high wetness indirectly corroborates the cold conditions seen in the tree-ring-based summer temperature for 1109 CE.

### Documentary evidence of societal impact and response in Western Europe

Diverse societal impacts and responses to these weather conditions are revealed by our sources, starting in 1109 CE. In France, Belgium and England, persistent wet summer and autumn weather reduced crop yields in this year. For France, Orderic Vitalis reports in the *Historia Ecclesiastica*  how excessive rainfall “*drowned the crops, the barrenness of the earth cried aloud, and the grape harvest was an almost total failure*” (Table S2). Similarly, the *Life of Blessed Tiron* describe how “*floods of rain*” in Northern and Western France rendered soils unproductive (Table S2). Crop shortages led to severe food price inflation in Burgundy (France), where wheat prices doubled between the winter of 1108/1109 and July 1109 CE according to *The Chronicle of Saint-Pierre-le-Vif de Sens* (Table S2). Although the text does not elaborate upon causation, as is characteristic of many annalistic and chronicle sources^46^, sharp food price increases often followed harvest shortfalls, compounded by grain hoarding and speculation by crop-owners seeking to maximize profits^47^. Withholding of grain supplies and speculation were observed, for example, after the back-to-back harvest failures of 1257/1258, 1315/1316/1317 and 1351/1352 CE^5,47–50^. The *Annals of Inisfallen* also provide evidence of societal coping mechanisms, demonstrating how these were contingent upon prevailing belief systems, in describing for Ireland how *“fastings and abstinence were observed and alms given to God”* so that the “*heavy rain and bad weather in the summer and autumn might be dispelled”* (Table S2).

**Figure 4:**
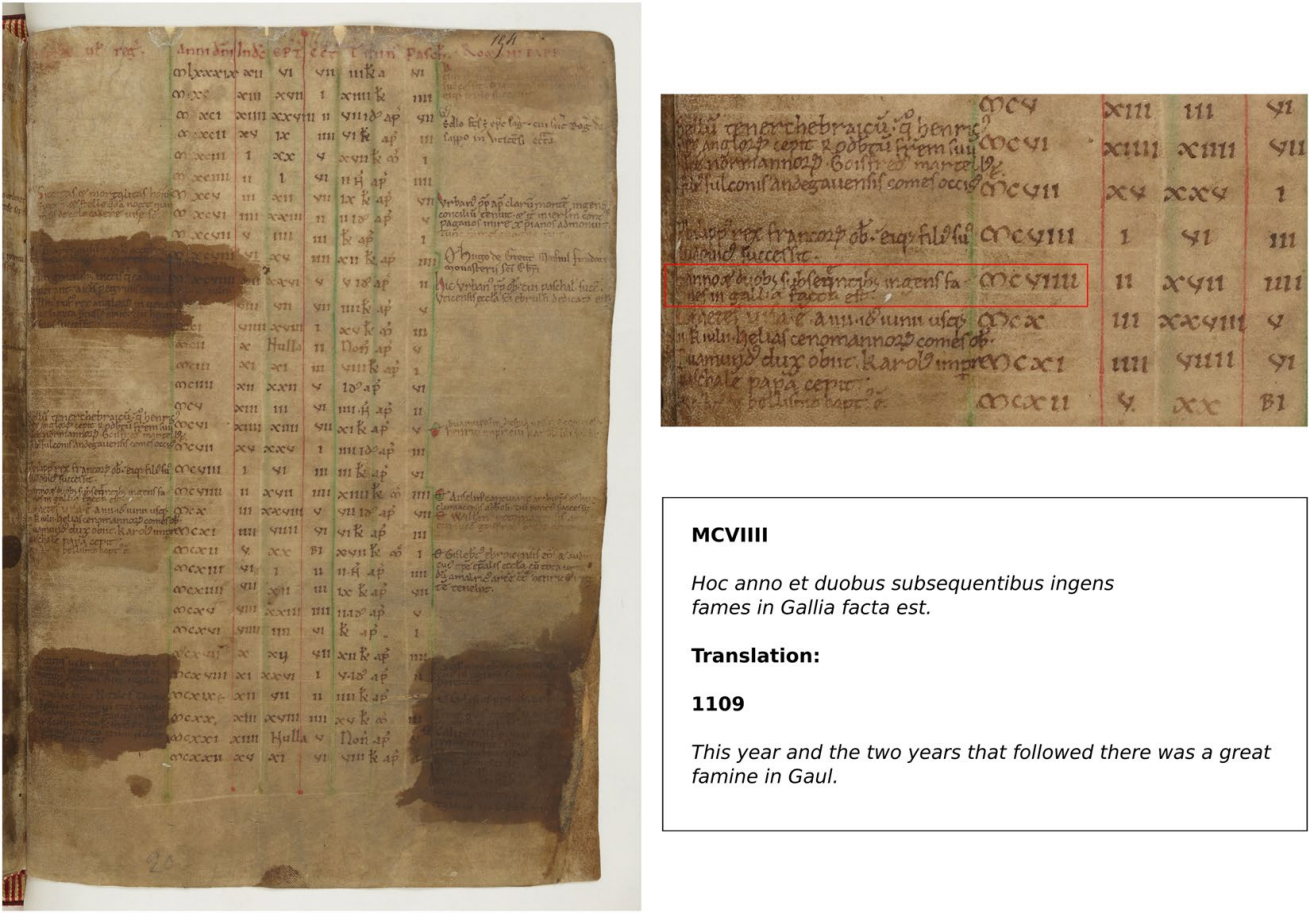
Original manuscript of the *Annals of Saint Evroult*, Normandy, France (source: *Annales Uticenses. Bibliothèque Nationale de France, Paris, Ms Latin 10062, f154r*). The manuscript displays a paschal table, also known as an Easter table, used to determine for successive years the changing dates on which Easter fell, one of the most important days in the liturgical calendar. On the margins of the Easter table, several brief historical notes were added, providing information mostly about king’s reigns, as well as the succession of abbots, bishops and Popes. The entry for the year 1109 CE, however, records a major famine in France lasting three years. This note was written at the abbey of Saint Evroult by a contemporary witness, the monk Orderic Vitalis (1075–1141/1143 CE). Orderic Vitalis is one of the most famous scribes of the 12^th^ century. He is predominantly known as the author of the *Historia Ecclesiastica*, in which he provides a more detailed and lengthy account of the famine of 1109–1111 CE (Table S2).

The assembled evidence suggests that the subsistence difficulties, which began in 1109, deepened into famine in several regions of western Europe, with particular severity in the kingdom of France (Fig. 4, Table S2). The *Chronicle of Morigny* (Etampes, Île de France) thus reports for 1109–10 CE that “*all of Gaul suffered from a severe famine and for seven continuous years the lack of everything required to maintain life lingered on, and thus killed off many people and reduced countless numbers of rich people to poverty*”. With its echoes of Joseph’s seven-year famine in Egypt as described in *Genesis*, we cannot take too literally the stated duration of the famine. Nevertheless, the chronicler’s intention is to underscore its uncommon severity. In summary, our exegesis points to onset of subsistence crises in 1109 CE, verging on or transitioning into famine in some regions in 1110 and persisting until 1111 CE. Notably, Orderic Vitalis dedicated two entries of his chronicle to famine. The first, pertaining to 1109 CE, noted that *“where both Ceres and Bacchus* [i.e., the Roman gods of cereals and wines, respectively] *failed, terrible famine decimated human beings everywhere*”, while the other, pertaining to 1110 CE, reported how “*for three consecutive years, from the second to the fourth indiction* [i.e., 1109 to 1111 CE]*, there was a terrible famine in France, and great numbers of folk were seriously weakened”*. Subsistence crises are also reported for Catalonia (Spain) in 1110 and 1111 CE^51^, and implied to have affected Ireland and England (Table S2). The limited number of meteorological observations or information related to subsistence crises in sources surveyed from the Holy Roman Empire (i.e., present-day Germany, Austria, and Italy), precludes any inference on societal consequences in these regions for the time being.

By reducing food availability, the climatic perturbations induced by the 1108–1110 eruptions may have initiated the preconditions necessary for famine. Importantly, however, subsistence crisis and famines arise from multiple, often interacting, influences occurring on a range of temporal scales, such as extreme weather coinciding with destructive military activities (e.g., warfare involving scorched earth tactics), burdensome economic policies (e.g. taxation) or rapid demographic changes^47^. The *Peterborough Chronicle* provides a valuable illustration of this point in supplying an explicit qualification of multi-causality that is rarely found in High Medieval sources. It reports that 1110 CE *“was a very disastrous year … through bad weather by which the earth-crops were badly damaged, and tree-crops all well-nigh ruined all over this land”*, but argues as well that the *“disastrous year”* also resulted from the burden of *“… the tax which the king took for his daughter’s gift …”* for her marriage to the emperor. It is also noteworthy that although the crisis that struck Western Europe between 1109 and 1111 CE was acute, at least 9 other major famines can be identified for the late 11^th^ and 12^th^ centuries, namely in 1093–1095, 1098–1099, 1124–1126, 1145–1147, 1162–1163, 1175–1177, 1181–1182, 1190–1192 and 1195–1198 CE, of which only two (1181-1182 and 1190-1192) closely coincide with known substantial volcanic eruptions, thereby highlighting that volcanically-induced climatic perturbations were not always the primary cause of famines in the 12^th^ century.

### Conclusions and perspectives

The new evidence for a stratospheric aerosol veil in 1110 CE on the basis of a contemporary observation of a dark total lunar eclipse allows an independent validation of the accuracy of the NS1–2011 timescale for the 12^th^ century. In this paper, we also document a mean NH cooling of about 1 °C in 1109 CE, an anomaly that is comparable to that observed following major eruptions in 1816, 1601, 1453, 1258/1259, and 536 CE, as well as a severe subsistence crisis in Western Europe that was likely preconditioned by extreme weather. We therefore posit that these anomalies may result from the cumulative climatic effects of several closely-spaced volcanic eruptions that occurred between 1108 and 1110 CE.

The eruptions of 1108–1110 CE therefore constitute, along with 536/40, 1453/58, and 1809/15 CE, a further example of a climatically and societally impactful cluster of explosive eruptions. But significant differences also exist in our case, given the close timing of this proposed cluster. The climatic impact of this 12^th^ century cluster may thus be less persistent than the decadal-scale cooling of the “double events” of 536/40, 1453/58, and 1809/15 CE that are separated by 5 to 6 years^52^.

Finally, our study provides an important example of the challenges frequently encountered in reconciling ice-core volcanic signals from multiple cores. It emphasizes the likelihood that some episodes of contiguous volcanic sulphate deposition in ice cores may result from more than one eruption, as recently demonstrated for the 1259 CE Antarctic signal^53^. A major ongoing challenge is thus to identify cases in which volcanic sulphate signals may represent the contribution of multiple events (e.g., where a bipolar volcanic signal results from two or more extratropical NH and SH events versus a single tropical event), which may produce meaningfully different climatic responses. A combination of evidence and methodologies is essential here. Documentary evidence such as that detailing the August 1108 CE eruption of Asama is advantageous in pinpointing the precise location, timing and, particularly when combined with geological evidence, magnitude of historical eruptions. However, magnitude (explosiveness) is not a direct measure of climatic forcing potential, and petrological evidence may help determine the sulphate output from Asama 1108 CE. We also encourage the search for and characterization of tephra in the Greenland and Antarctic ice cores at this time as a means of fingerprinting the contribution of specific volcanoes. Such measures can not only test and further refine our proposed scenarios, which is essential, but also yet further scenarios that are conceivable when considering variable sulphate transport times to both poles and remaining chronological uncertainties in the NS1–2011 and WD2014 time-scales.

## Material and Methods

### Analysis of contemporary historical sources

More than 130 medieval documentary sources from the 12^th^ century were extensively searched for references to possible climatic impacts of the “forgotten”, early 12^th^ century volcanic eruption cluster. Contemporary sources are mostly found in compilations of medieval texts edited in the series of the (i) *Monumenta Germaniae Historica*, (ii) *Rerum Britannicarum Medii Ævi scriptores* and *Annales Monastici* (often simply known as “The Rolls Series”), (iii) *Recueil des historiens des Gaules et de la France* as well as in the (iv) *Rerum Italicarum scriptores*. In addition, several annals, chronicles and vitae have been surveyed; these included, among others, the *Peterborough Chronicle* (part of the *Anglo-Saxon Chronicle*), the ecclesiastical history of Orderic Vitalis, the chronicle of Saint-Pierre-le-Vif de Sens, the chronicle of Morigny and the life of Blessed Bernard of Tiron (see Table S2). Original sources were mostly written in Latin and to a lesser extent in vernacular (i.e. Old English) and Middle Irish (Gaelic).

### Assessing coloration of lunar eclipses

Particular attention was focused on optical phenomena suggestive of the presence of volcanic aerosols in the stratosphere^21^, such as the dimming of the Sun or dark total lunar eclipses. Concerns regarding remaining inconsistencies in the dating of volcanic events for the 1450 s and 1690s, stimulated by suggested mismatches between volcanic forcing data and responses in tree-ring-based temperature reconstructions^54,55^, motivate a search for additional validation of the newly re-dated ice core volcanic horizons using independent and securely dated historical evidence of lunar brightness.

During a total lunar eclipse, sunlight incident on the Earth limbs is absorbed and then scattered. The green to violet portion of the spectrum is most affected while most of the orange to red light is transmitted through the atmosphere. The remaining light is refracted by the atmosphere into the Earth shadow, projecting indirect, reddish light on to the Moon. This gives the fully-eclipsed Moon its characteristic copper to deep-red color. In the presence of volcanic aerosol in the stratosphere, however, refraction and scattering of sunlight into the Earth shadow is greatly diminished, hence the eclipsed Moon is darkened. In extreme cases, it can become practically invisible to naked-eye observation^23,24^. Here, the reliability of eclipse observations was assessed using NASA’s *Five Millennia Catalog of Lunar Eclipses* covering the period 1999 BCE – CE 3000^25^. The darkness of the Moon was rated according to the Danjon scale^56^ ranging from L = 0 (very dark, indicating a high turbidity of the stratosphere) to L = 4 (very bright, copper-red or orange, indicating low stratospheric turbidity)^17^. We gave greatest weight to first-hand information derived from contemporary sources, and dates in chronicles were screened systematically for errors by cross-checking information with other manuscripts (Table S1 and Table S2). Care was taken to identify duplicated reports. Duplicates refer to historical material for which the author did not witness the event described but rather copied the information from another source. The content of duplicated reports is therefore not original. Frequent duplication occurred in the Middle Age due to the shared ancestry and common source material of many surviving texts. All duplicates that were identified are listed in Table S1 and S2 but were not used for the analyses.

### Tree-ring based reconstruction of NH summer temperatures

#### NH Reconstruction

To quantify the NH summer cooling induced by the 1108–1110 CE eruptions, we used an updated version of the *NVOLC* reconstruction (*NVOLC* v2) published by Guillet *et al*. (5). *NVOLC* v2 consists of 25 proxy records (13 tree-ring width and 12 maximum latewood density chronologies). The first version of *NVOLC* was initially designed to quantify the NH summer cooling induced by the 1257 CE Samalas eruption. To estimate the changes in temperature observed after the 1257 CE eruption in Greenland – where no millenium-long tree-ring chronology is available - 3 annually resolved stable isotope series from Greenland, namely the GRIP, Crete and DYE3 records, were incorporated to the *NVOLC* dataset. However these records have been dated using the GICC05 timescale. While the chronological error of the GICC05 timescale is small around the time of the 1257 CE Samalas eruption, Sigl *et al*. (6) have shown that for earlier years such as 1108–1110 CE, a more substantial level of noise may be added. We therefore excluded these records from the *NVOLC* v2 network and recomputed the NH JJA reconstruction.

A principal component regression (PCR) approach was chosen to reconstruct NH JJA temperature anomalies (wrt 1961–1990). The bootstrap method was combined with the PCR to estimate the skill of the reconstruction and compute confidence intervals of reconstructed JJA temperatures. To gradually adjust to the changing number of records available back in time, we combined the PCR with a nested approach. *NVOLC* v2 reconstruction is based on 23 subsets of tree-ring chronologies or nests. The earliest and most recent nests span the periods 500–551 and 1992–2000 CE, respectively. The most replicated nest is composed of 25 chronologies and spans the period 1230–1972 CE (see Table S4). The nested PCR was computed as follows:

For each nest, a principal component analysis was calculated on the proxy predictors and the first *n* principal components (PCs) with eigenvalues >1 were retained as predictors to develop a multiple linear regression model. The regression models were all calibrated on JJA temperature obtained from the Berkeley Earth Surface (BEST) dataset over the period 1805–1972 CE. For each nested regression model, a split calibration–verification procedure was repeated 1,000 times using the bootstrap method to assess the robustness of the transfer function and 1,000 reconstructions were generated. The final reconstruction of each nest is the median of the 1,000 realizations and is given with its 95% bootstrap confidence interval (Fig. 3). For each nest, the coefficient of determination (r2 for the calibration and R2 for the verification periods), RE (reduction of error) and CE (coefficient of efficiency) statistics were calculated. These statistics are illustrated with their 2.5 and 97.5 percentiles in Table S4. The *NVOLC* v2 reconstruction (500–2000 CE) is obtained by appending together the 23 reconstructions, with their mean and variance adjusted to be the same as the most replicated nest. Finally, to quantify the reconstructed cooling within a context of climate variability prevailing at the time of major volcanic eruptions, we filtered the *NVOLC* v2 series with a 31-yr running mean (*NVOLC_filt* v2). For example, in the case of the year 1109 CE, a background was calculated by averaging the window 1094–1108 CE and 1110–1124 CE. The anomaly is then created by subtracting this background from the 1109 CE reconstructed temperature.

#### Climate Field Reconstruction

To estimate regional variability of summer cooling induced the 1108–1110 CE eruptions, we developed a climate field reconstruction of extratropical Northern Hemisphere summer temperatures spanning the last 1500 years. The target field (predictand) used for the reconstruction is the BEST JJA gridded temperature dataset (1°x1° latitude-longitude grid). The Northern Hemisphere was divided into 11 regions based on the spatial distribution of the 25 chronologies and their correlation (Fig. S1, Table S3). A minimum coefficient correlation (r) threshold above 0.3 (p < 0.05) over the common period of the chronologies was used to cluster the tree-ring records in the same region. For each region, the number of chronologies ranges between 1 (Quebec, Central Europe, Siberia - Taymir, Siberia - Yakutia, and China - Qilian Mountains) and 5 (Western Europe).

For cluster with several chronologies (*n* ≥ 2), we applied a nested PCR to reconstruct the JJA surface temperatures anomaly field. For clusters with only one chronology, we used an ordinary least square regression. The performance and temporal stability of reconstruction models over the 1901–1972 period common to all chronologies was assessed for each grid point through a split-sample calibration and verification approach repeated 1000 times using the bootstrap method. For each grid point the skills of the reconstruction was assessed using the r2, R2, RE and CE statistics (Fig. S2). Based on these statistics, a total of 3486 grid points (CE > 0.1) in the Northern Hemisphere have been reconstructed back to 500 CE, with a minimum number of 52 grid points (CE > 0.1) for the Indigirka cluster and maximum of 918 grid points (CE > 0.1) for the Siberia - Polar Ural cluster.

## Supplementary information

Supplementary Information.

Table S1 and Table S2.

## Data Availability

The data used to perform our analysis as well as our results have been uploaded to Zenodo and are accessible using the following link: 10.5281/zenodo.3724674
