# Supplementary material for: Climatic and societal impacts of a “forgotten” cluster of volcanic eruptions in 1108-1110 CE
Source: Sci Rep. 2020 Apr 21;10:6715. doi: 10.1038/s41598-020-63339-3 (PMC7174372; doi:10.1038/s41598-020-63339-3)

Climatic and societal impacts of a “forgotten” cluster of volcanic eruptions in 1108-1110 CE

Sébastien Guillet[1,*], Christophe Corona[2], Francis Ludlow[3], Clive Oppenheimer[4], Markus Stoffel[1,5,6,]

[1] Climatic Change Impacts and Risks in the Anthropocene (C-CIA), Institute for Environmental Sciences, University of Geneva, 66 Boulevard Carl Vogt, CH-1205 Geneva, Switzerland.

[2] Geolab, Université Clermont Auvergne, CNRS, F-63000 Clermont-Ferrand, France.

[3] Department of History, School of Histories & Humanities, Trinity College, Dublin, D2, Ireland

[4] Department of Geography, University of Cambridge, Downing Place, Cambridge CB2 3EN, United Kingdom.

[5] Department of Earth Sciences, University of Geneva, Rue des Maraîchers 13, CH-1205 Geneva, Switzerland.

[6] Department F.-A. Forel for Environmental and Aquatic Sciences, University of Geneva, 66 Boulevard Carl Vogt, CH-1205 Geneva, Switzerland.

* Corresponding author: sebastien.guillet@unige.ch

Supplementary Information

Texts, tables and figures – Supplementary Information

Table S1. Lunar eclipses reported in European historical archives between 1100 and 1120 CE Refer to: "Guillet_et_al_SciReports2020_Supp_Info_Table_S1_S2_Historical_Sources.xlsx"

Text S1. The total lunar eclipses of 1110 CE.

Table S2. Documentary sources describing the weather anomalies and unusual optical phenomena observed in Europe in the aftermath of the 1108-1110 CE eruptions. Refer to the file: "Guillet_et_al_SciReports2020_Supp_Info_Table_S1_S2_Historical_Sources.xlsx"

Table S3. Tree-ring chronologies used in this study for the reconstruction of Northern hemisphere (NH) JJA temperature anomalies for the period 500–2000 CE.

Figure S1. Location map of the proxy data used in this study.

Table S4. Calibration and verification statistics for the *NVOLC* v2 reconstruction for each nest. nchron - Number of chronologies, p - Number of principal components; R2b - Median value of R2b (1000 bootstrap iterations) calculated over the calibration period; r2b - Median value of r2b (1000 bootstrap iterations) calculated over the verification period; REb - Median value of REb (Reduction of Error, 1000 bootstrap iterations) calculated over the calibration period; CEb - Median value of CEb (Coefficient of Efficiency, 1000 bootstrap iterations) calculated over the calibration period. Numbers in brackets refer to the 2.5th and 97.5th percentiles of the R2b, r2b, REb, and CEb distributions.

Figure S2. Maps of R^2, r^2, RE and CE obtained for the spatial reconstruction.

Table S5. Frost rings and light-rings observed in the tree-ring chronologies of the Northern hemisphere in the aftermath of the 1108-1110, 1257 Samalas, 1453 unknown, 1601 Huaynaputina and 1816 CE Tambora eruptions.

Figure S3. Persistence of climatic anomalies in the Northern hemisphere following the 1108-1110, 1257 Samalas and 1816 CE Tambora eruptions.

Figure S4. Climatic anomalies and societal impacts reported in European historical archives during the summers of 1109-1110, 1174 and 1195-1196 CE.

Figure S5. Number of narrative sources providing weather reports per year and season over the 12th century. Clear peaks are observed following the severe drought of 1137, the cold winter of 1149/1150 and the wet and cold summers of 1109, 1151, 1174, 1195-1196 CE.

Figure S6. Tree-ring based reconstructions of the warm season Palmer Drought Severity index for Europe for the boreal summer of 1109 CE (Cook et al., 2015), expressed as anomalies from a contemporary reference period (1094–1124 CE).

Text S1. The Total Lunar Eclipses of 1110 CE

1. The visibility of the lunar eclipse of May 5, 1110

The total lunar eclipse that occurred on the 5th of May 1110 was visible across Europe, the Middle East and Africa (Espenak & Meeus, 2009, Figure 1).

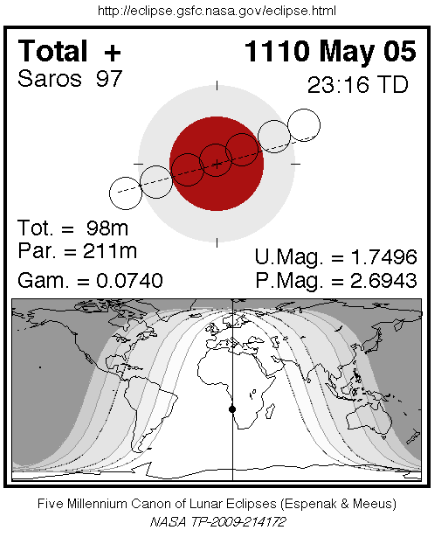

Figure 1. The total lunar eclipse of the 05 May 1110: Visibility map (Source: Espenak & Meeus, 2009)

To our knowledge, Europe is the only region for which accounts describing this particular lunar eclipse have so far been retrieved.

2. Historical sources having recorded the total lunar eclipses of May 5, 1110

Observation of the eclipse in Europe:

A total of 10 monastic chronicles and annals originating from Belgium, England, France and Germany recorded the total lunar eclipses of May 5, 1110 (see Table S2 for more details), namely the:

- *The Annales Formoselenses* (Saint Peter's Abbey, Ghent/Voormezeele, Belgium)
- *The Peterborough Chronicle* (Monastery of Peterborough, England)
- *The Chronica Majora (Saint Alban, England)*
- *The Flores Historiarum (Saint Alban/Westminster, England)*
- *The Annals of Waverley* (Waverley abbey, Farnham, England)
- *The Annals of the Priory of Bermondsey* (Bermondsey, England)
- *Ex Historiae Francicae Fragmento* (Saint-Benoit-sur-Loire, France)
- *The Chronicle of Saint-Maixent* (Saint-Maixent-L'Ecole, France)
- *The Annales Hildesheimenses* (Hildesheim, Germany)
- *The Annalista Saxo* (Nienburg, Germany)

Of the ten historical sources listed above, six provided original (i.e., independent) information about the total lunar eclipse of May 5, 1110 (see Table S2). The records from four sources were discarded, namely the *Chronica Majora*, the *Flores Historiarum*, the *Annals of Waverley* and the *Annals of the Priory of Bermondsey*, since most of the information referring to the total lunar eclipse are derived from the *Peterborough Chronicle* and are consequently not original.

The *Peterborough Chronicle* is the only source providing information about the color of the eclipse. Other sources merely mention the eclipse, without providing any detail and are therefore not useful to detect the presence of volcanic aerosols in the stratosphere. Several factors may explain why only a limited number of accounts carefully described the total lunar eclipse of May 5, 1110:

1. First of all, it must be stressed that interest in celestial phenomena among monastic chroniclers evidently varied very much from one individual to another, and the purpose of the sources in question was not to provide a dedicated astronomical record. While several writers did seem to have a special interest in astronomical matters, recording many eclipses and describing their appearance, others passed over these events in silence or merely stated that they were happening (Stephenson, 1997).

2. The lack of detailed description of the obscuration of the moon can also be explained by unfavorable weather or persistent clouds interfering with the observer's attempts to view the eclipse.

3. Finally it must be added that a comet was sighted just one month after the lunar eclipse. This comet was observed worldwide in China, Korea, Japan, the Middle East and Europe (Kronk, 1999). In Europe, the 1110 comet is reported in the monastic chronicles from Belgium, England, Italy, France, Germany and Scotland. It is interesting to note that the descriptions of the 1110 comet that have reached us are numerous and quite detailed (Kronk, 1999; see also Table S2). Chroniclers and annalists often noted the number of days during which the comet was visible, its location in the sky and its appearance (see Table S2). Such a level of detail is not present for the total lunar eclipse of May 5, 1110, except for the *Peterborough Chronicle*. Comets were frequently considered in Medieval Europe as ominous signs presaging misfortune, war, plague and mortality of men (Schechner, 1999). The treatise on the comet of 1299 (Thorndike, 1945) written by Peter of Limoges that we have reproduced below illustrates quite well the fear caused comets in medieval Europe:

> "In the year of the lord 1299, before the end of January and all through February and for some days in March, appeared a comet of moderate size with a long tail for the size of its body, of a color somewhat bordering on dark blue, from which I infer that it was the variety of comet known as Lord Ascone, which in Arabic and In Hebrew means the same as Lord of Death in Latin. [...] These things being so, I say according to the doctrine of judicial astrology that a comet of this sort signifies wars, conflicts, future diseases or scab and itch, mortality of men and beasts from corruption of the air, many violent winds, great earthquakes in certain parts of the earth, excessive rains, vehement cold in winter time, failure and scarcity of victuals, rotting of crops and of the fruit of trees, and destruction of the same. And because the phenomenon has lasted for many days, it indicates that its sequels will be of long duration. […]"
>
> Thorndyke, 1945, Isis 36 (1):3-6

Therefore these rare celestial events engendered anxiety and it is possible that annalists and chroniclers that witnessed both the total lunar eclipse of May 05, 1110, and the

comet chose to focus their attention on the latter rather than on the total lunar eclipse, which is a celestial phenomenon observed more frequently, the timing of which was even known to be potentially predictable from the early medieval period.

Observation of the eclipse in Islamic Spain, Egypt, Iraq and Syria

Said and Stephenson (1997) carefully investigated lunar eclipses reported in Arabic chronicles. The lunar eclipse reports they found mostly come from al-Andalus (Islamic Spain), Egypt, Iraq, and Syria. They mainly cover the date range from A.D. 867 to 1520, with additions from 1674 to 1791. Unfortunately, they couldn't find any source reporting the eclipse that happened on May 1110. There is, indeed, a gap of about 150 years between 969 and 1117 in the lunar eclipses catalogue they compiled.

Lunar obscurations were also observed in the Middle East by Christians who participated to the Crusades and later referred to these events in European chronicles. For this reason, we also investigated all chronicles edited in the *Recueil des Historiens des Croisades* (*Collection of the Historians of the Crusades*), a major collection of medieval documents, which were edited and published in the 19th century. Although we could find in the *Chronicle of Fulcher of Chartres* a description of the total lunar eclipse that occurred on the 15th of June 1117 (Table S2), regrettably, we could not locate any mention of 1110 total lunar eclipse.

Observation of the eclipse in China, Japan and Korea

The total lunar eclipse of May 5, 1110, was not visible in Japan and Korea (Figure 1) and was consequently not reported in Japanese and Korean sources. The eclipse only was partly visible in China. It is recorded as follows in the astronomical treatise of the *Songshi,* the official history of the Song dynasty (A.D. 960 – 1279) (Xu et al., 2000):

AD 1110 May 6

"Emperor Huizong of Song, 3rd year of Daguan reign period, 4th month, day *jiashen*; the Moon was totally eclipsed. "

[Song Shi - Tianwen zhi] wu ch. 52

Regrettably the account provides no information about the brightness of the eclipse, preventing us from drawing any conclusion regarding the turbidity of the stratosphere in this region.

3. The total lunar eclipse of October 29, 1110

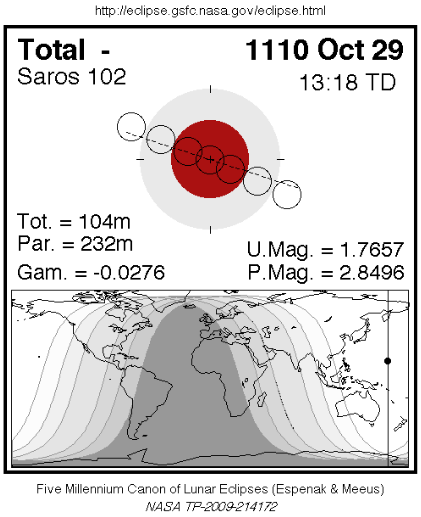

Figure 2. The total lunar eclipse of the 29 October 1110: Visibility map (Source: Espenak & Meeus, 2009)

Another total lunar eclipse occurred on 29 October, 1110. While the phase of totality was not visible in Europe and in the Middle East, it was visible in China, Korea and Japan (Espenak & Meeus, 2009). We surveyed the catalog published by Xu et al. (2000) to determine whether the eclipse was recorded in east-Asian sources and whether they reported the brightness and color of the eclipse. Xu et al. (2000) did not recover any account reporting the eclipse in Japanese and Korean sources but did in the Chinese astronomical treatise of the *Songshi the following report:*

> AD 1110 Oct 29
>
> "Emperor Huizong of Song, 4th year of the Daguan reign period, 9th month day *gengchen*; the Moon was totally eclipsed."
>
> [Song Shi - Tianwen zhi] wu ch. 52

As with the total lunar eclipse of May 5, 1110, the account is very brief, giving no more that the day of occurrence. We also note that Lee et al. (2016) recently reported Korean sources that do refer to the total lunar eclipse of October 29, 1110, but without providing a full description of the account.

4. On the value of East-Asian and Arabic reports of lunar eclipse for estimating stratospheric turbidity

It is worth noting that the accounts of lunar eclipses from East Asia that have reached us rarely provide information about the color of lunar eclipses, which limits their use for the detection of volcanic aerosols in the stratosphere. This is especially true for Korean and Chinese sources (Stephenson, 2008).

Japanese records of eclipse tend to be more detailed and sometimes mention, although very rarely, the coopery hue typically observed during a lunar eclipse:

AD 1085 Sep 6

"2nd year of the Otoku reign period, 8th month, 15th day of the month, day *bingzi*. During the hour of *hai* the Moon was totally eclipsed. The moon was reddened and without light, and the sky was dark."

[Fusō ryaku ki] ch. 30

Similar comments can be made about Arabic annals and chronicles. In a paper published in 1997 in *Bulletin of the School of Oriental and African Studies,* Stephenson and Said discussed the limited interest of Arabic chroniclers in describing the color of the moon during an eclipse:

"On account of dispersion of light in the Earth's atmosphere the totally eclipsed moon often has a deep red colour. However, in medieval Islamic history, there seems to be no definite instance of the moon being described in this way at an eclipse. The earliest reliable observation of this kind is noted by the eighteenth-century historian al-'Umari of Mosul, who in reporting the total eclipse of A.D. 1772 remarks that the moon became like "copper red hue"."

5. References

Espenak, F., & Meeus, J. (2009). *Five Millennium Catalog of Lunar Eclipses: -1999 to +3000* (NASA).

Kronk, G. W. (1999). *Cometography: a catalog of comets* (Vol. 1). Cambridge ; New York: Cambridge University Press.

Lee, K.-W., Mihn, B.-H., Ahn, Y. S., & Ahn, S.-H. (2016). Analysis of the Lunar Eclipse Records from the Goryeosa. *Journal of The Korean Astronomical Society*, *49*(4), 163–173. https://doi.org/10.5303/JKAS.2016.49.4.163

Schechner, S. J. (1999). *Comets, popular culture, and the birth of modern cosmology* (3. print., 1. paperback print). Princeton, NJ: Princeton Univ. Press.

Stephenson, F. R. (1997). *Historical Eclipses And Earth's Rotation*. Cambridge: Cambridge University Press. https://doi.org/10.1017/CBO9780511525186

Stephenson, F. R., & Said S. Said. (1997). Records of Lunar Eclipses in Medieval Arabic Chronicles. *Bulletin of the School of Oriental and African Studies, University of London*, *60*(1), 1–34.

Thorndike, L. (1945). Peter of Limoges on the Comet of 1299. *Isis*, *36*(1), 3–6. https://doi.org/10.1086/347896

Xu, Z., Pankenier, D. W., & Jiang, Y. (2000). *East Asian archaeoastronomy: historical records of astronomical observations of China, Japan and Korea*. Amsterdam, Netherlands : Amherst, N.Y: Published on behalf of the Earth Space Institute by Gordon and Breach Science Publishers ; Cambria Press.

Table S3. Tree-ring chronologies used in this study for the reconstruction of Northern hemisphere (NH) JJA temperature anomalies for the period 500–2000.

ID	Cluster name	Proxy	Tree ring chronologies	Country	Latitude	Longitude	Species	Parameter	Chronology type	Period	References
1	Alaska - Northern Canada	Tree ring	Firth River	United States	68.8	-142.4	*Picea glauca* (Moench) Voss	MXD	SF-RCS	1073-2002	*Anchukaitis et al., 2013*
		Tree ring	Seward Composite	United States	65.16	-162.25	*Picea glauca* (Moench) Voss	TRW	STD	978-2002	*D'Arrigo et al., 2006*
		Tree ring	Coastal Alaska (GOA)	United States	60.5	-146	*Tsuga mertensiana* (Bong.) Carrière	TRW	RCS	713-2003	*D'Arrigo et al., 2006*
		Tree ring	Campbell Dolomites B	Canada	68.3	-133.3	*Picea glauca* (Moench) Voss	MXD	RCS	1175-1992	*Schweingruber, ITRDB; Schneider et al., 2015*
2	British Columbia	Tree ring	Athabasca - Icefields	Canada	52.3	-117.3	*Picea engelmannii*	MXD	RCS	1072-1994	*Luckman et al., 2005*
		Tree ring	Coastal British Columbia (CWD)	Canada	52	-126	*Tsuga mertensiana* (Bong.) Carrière	TRW	STD	1094-2010	*Pitman et al., 2012*
3	Quebec	Tree ring	Quebec - Strec	Canada	57.3	-74	*Picea mariana* (Mill.) B.S.P	TRW	RCS	910-2011	*Gennaretti et al., 2014*
4	Western Europe	Tree ring	French Alps - Composite (Merveilles - Nevache Granges - Fressinière Grand-Bois)	France	44	7	*Larix decidua* Mill.	TRW	SF-RCS	751-2007	*Corona et al., 2010; This study*
		Tree ring	Lauenen	Switzerland	46.4	7.3	*Picea abies (L.)* H. Karst	MXD	RCS	982-1976	*Schweingruber et al., 1988*
		Tree ring	Lötschental	Switzerland	47.5	7.5	*Larix decidua* Mill.	MXD	RCS	735-2004	*Büntgen et al., 2006*
		Tree ring	Tyrol	Austria	47.5	12.5	*Picea abies (L.)* H. Karst	MXD	RCS	1047-2003	*Esper et al., 2007*
		Tree ring	Pyrenees	Spain	42.5	2.5	*Pinus uncinata* Ram	MXD	RCS	1044-2005	*Büntgen et al., 2008*
5	Central Europe	Tree ring	Tatra	Slovakia	48-49	19-20	*Larix decidua* Mill.	TRW	RCS	963-2011	*Buntgen et al., 2013*
6	Scandinavia	Tree ring	Nscan	Finland-Sweden	67.5	22.5	*Pinus silvestris*	MXD	RCS	-181- 2006	*Esper et al., 2012*
		Tree ring	Torneträsk	Sweden	68.2	19.7	*Pinus silvestris*	MXD	RCS	441-2010	*Melvin et al., 2013*
		Tree ring	Jämtland	Sweden	63.5	15.5	*Pinus silvestris, Picea abies (L.)* H. Karst	MXD	RCS	1111-1978	*Schweingruber et al., 1988; Schneider et al., 2015*
		Tree ring	Forfjorddalen 2	Norway	68.8	15.73	*Pinus silvestris*	TRW	STD	877-1994	*Kirchhefer et al., 2000*
7	Siberia - Polar Ural	Tree ring	Polar Ural	Russia	66.9	65.6	*Larix sibirica* Ledeb, *Picea obovata* Ledeb	MXD	SF-RCS	778-2006	*Briffa et al., 2013*
		Tree ring	Yamal	Russia	67.32	69.54	*Larix sibirica* Ledeb	TRW	SF-RCS	-764-2005	*Briffa et al., 2013*
8	Siberia - Taymyr	Tree ring	Taymyr	Russia	72	102	*Larix gmelinii* Rupr.	TRW	RCS	-207-2003	*Naurzbaev et al., 2002 ; Briffa et al., 2008; Sidorova et al., 2010*
9	Siberia Yakutia	Tree ring	Indigirka - Yakutia	Russia	69-70	148-149	*Larix cajanderi* Mayr.	TRW	RCS	-929-2004	*Sidorova and Nauzbaerv, 2002; Sidorova 2003; Hughes et al., (in preparation)*
10	Central Asia Altai - Mongolia	Tree ring	Altai	Russia	50	88	*Larix sibirica* Ledeb	TRW	RCS	-359-2011	*Myglan et al., 2012*
		Tree ring	Altai	Russia	50	88	*Larix sibirica* Ledeb	MXD	RCS	556-2007	*Schneider et al., 2015*
		Tree ring	OZN	Mongolia	51.15	99.04	*Larix sibirica* Ledeb	TRW	SF-RCS	931-2005	*Davi et al., 2015*
11	China - Qilian Mountains	Tree ring	Qilian Mountains - HY	China	99.7	38.7	*Sabina przewalskii* Kom., *Picea crassifolia* Kom.	TRW	STD	670-2012	*Zhang et al., 2014*

Figure S1. Location map of the proxy data used in this study.

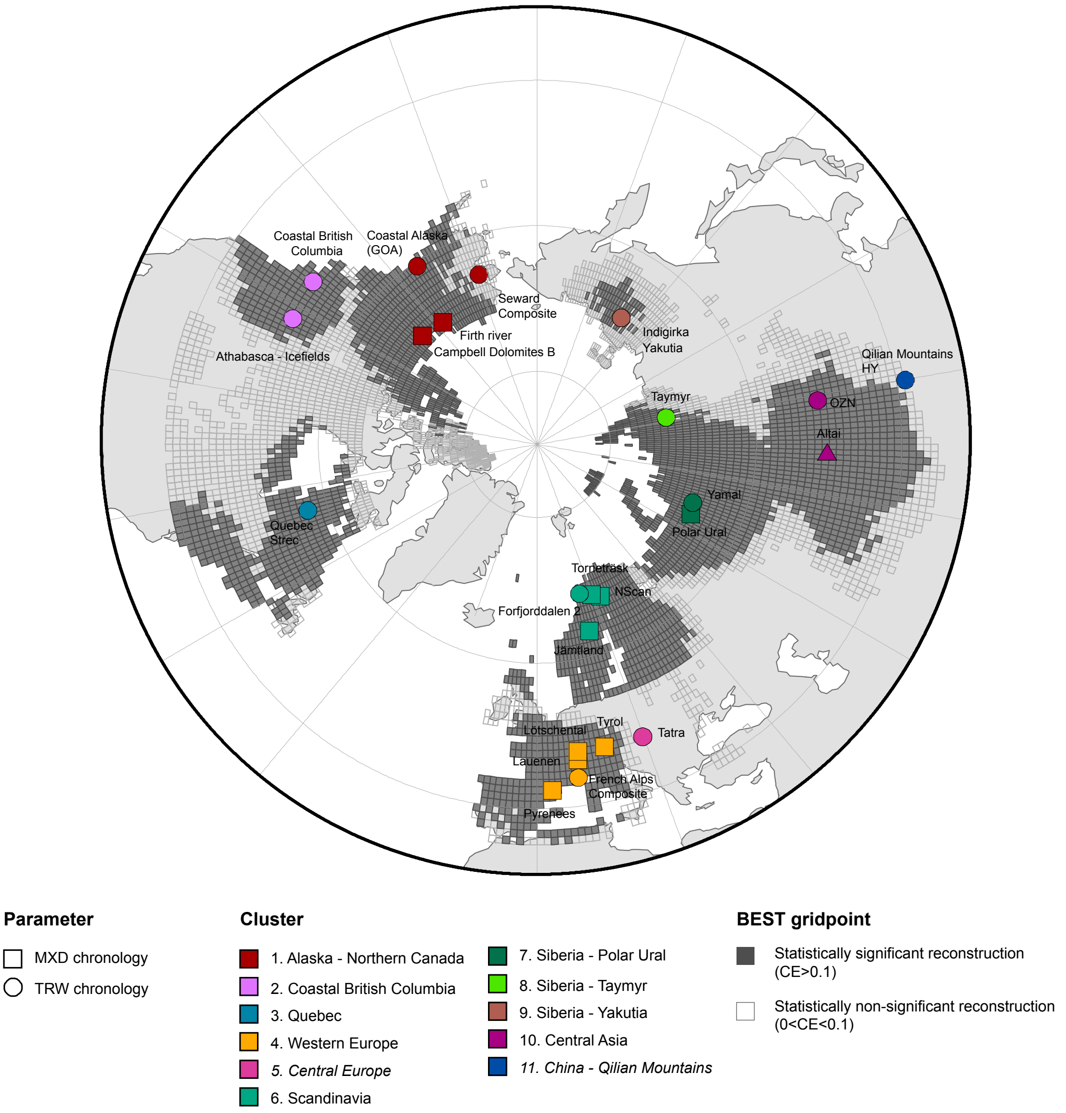

Table S4. Calibration and verification statistics for the *NVOLC* v2 reconstruction for each nest. nchron - Number of chronologies, p - Number of principal components; R2b - Median value of R2b (1000 bootstrap iterations) calculated over the calibration period; r2b - Median value of r2b (1000 bootstrap iterations) calculated over the verification period; REb - Median value of REb (Reduction of Error, 1000 bootstrap iterations) calculated over the calibration period; CEb - Median value of CEb (Coefficient of Efficiency, 1000 bootstrap iterations) calculated over the calibration period. Numbers in brackets refer to the 2.5th and 97.5th percentiles of the R2b, r2b, REb, and CEb distributions.

Nest - Period	500-551	551-556	556-670	670-713	713-743	743-865
nchron	6	6	7	8	9	10
p	3	3	4	7	4	6
R^2b	0.27[0.17-0.37]	0.27[0.14-0.37]	0.3[0.21-0.41]	0.34[0.22-0.47]	0.27[0.18-0.38]	0.38[0.25-0.47]
r^2b	0.23[-0.01-0.34]	0.23[0.07-0.37]	0.24[-0.03-0.35]	0.23[-0.17-0.37]	0.23[0.01-0.33]	0.29[-0.09-0.44]
REb	0.2[-0.01-0.33]	0.21[0.01-0.35]	0.23[-0.07-0.33]	0.21[-0.18-0.36]	0.22[-0.03-0.33]	0.27[-0.12-0.43]
CEb	0.21[0-0.34]	0.22[0.02-0.36]	0.24[-0.05-0.34]	0.22[-0.16-0.37]	0.23[-0.01-0.34]	0.28[-0.1-0.44]

Nest - Period	865-880	880-910	910-931	931-950	950-996	996-1044
nchron	11	12	13	14	15	16
p	6	3	4	4	9	3
R^2b	0.38[0.23-0.5]	0.39[0.27-0.53]	0.41[0.29-0.52]	0.41[0.27-0.53]	0.46[0.34-0.58]	0.4[0.31-0.5]
r^2b	0.3[0.06-0.47]	0.35[0.08-0.47]	0.35[0.1-0.5]	0.36[0.15-0.47]	0.35[0.08-0.49]	0.38[0.1-0.48]
Reb	0.29[-0.01-0.46]	0.32[0.05-0.46]	0.33[0.06-0.49]	0.34[0.09-0.46]	0.33[0.08-0.48]	0.36[0.05-0.46]
CEb	0.3[0-0.47]	0.33[0.06-0.46]	0.35[0.08-0.5]	0.35[0.1-0.47]	0.34[0.09-0.49]	0.37[0.06-0.47]

Nest - Period	1044-1047	1047-1073	1073-1111	1111-1140	1140-1170	1170-1221
nchron	17	18	19	20	21	22
p	3	3	6	5	5	3
R^2b	0.43[0.28-0.53]	0.4[0.28-0.48]	0.43[0.32-0.55]	0.41[0.3-0.54]	0.42[0.33-0.54]	0.4[0.3-0.49]
r^2b	0.39[0.12-0.51]	0.37[0.05-0.46]	0.37[-0.02-0.52]	0.38[0.06-0.5]	0.39[0.02-0.52]	0.37[0.14-0.49]
REb	0.37[0.03-0.5]	0.35[0-0.46]	0.36[-0.1-0.5]	0.35[0.02-0.48]	0.37[-0.07-0.5]	0.35[0.07-0.47]
CEb	0.38[0.04-0.51]	0.36[0.01-0.47]	0.37[-0.08-0.51]	0.36[0.04-0.48]	0.38[-0.05-0.51]	0.36[0.08-0.48]

Nest - Period	1221-1225	1225-1230	1230-1972	1972-1992	1992-2000
nchron	23	24	25	23	19
p	12	3	10	4	5
R^2b	0.51[0.4-0.65]	0.42[0.33-0.52]	0.47[0.35-0.57]	0.41[0.32-0.5]	0.44[0.32-0.54]
r^2b	0.36[0.08-0.54]	0.35[0.07-0.47]	0.36[0.13-0.5]	0.38[0.16-0.49]	0.37[0.12-0.52]
Reb	0.35[-0.04-0.54]	0.34[-0.04-0.46]	0.35[0.11-0.48]	0.36[0.06-0.48]	0.36[0.04-0.49]
CEb	0.36[-0.02-0.55]	0.35[-0.02-0.47]	0.36[0.13-0.49]	0.38[0.08-0.49]	0.37[0.05-0.5]

nchron - Number of chronologies

p - Number of principal components

Median value of R^2b (1000 boostrap iterations) calulated over the calibration period

Median value of r^2b (1000 boostrap iterations) calulated over the verification period

Median value of REb - Reduction of Error (1000 boostrap iterations) calulated over the calibration period

Median value of CEb - Coefficient of Efficiency (1000 boostrap iterations) calulated over the calibration period

Number in brackets refer to the 2.5th and 97.5th percentiles of the R^2b, r^2b, REb, and CEb distributions

Figure S2. Maps of R^2, r^2, RE and CE obtained for the spatial reconstruction.

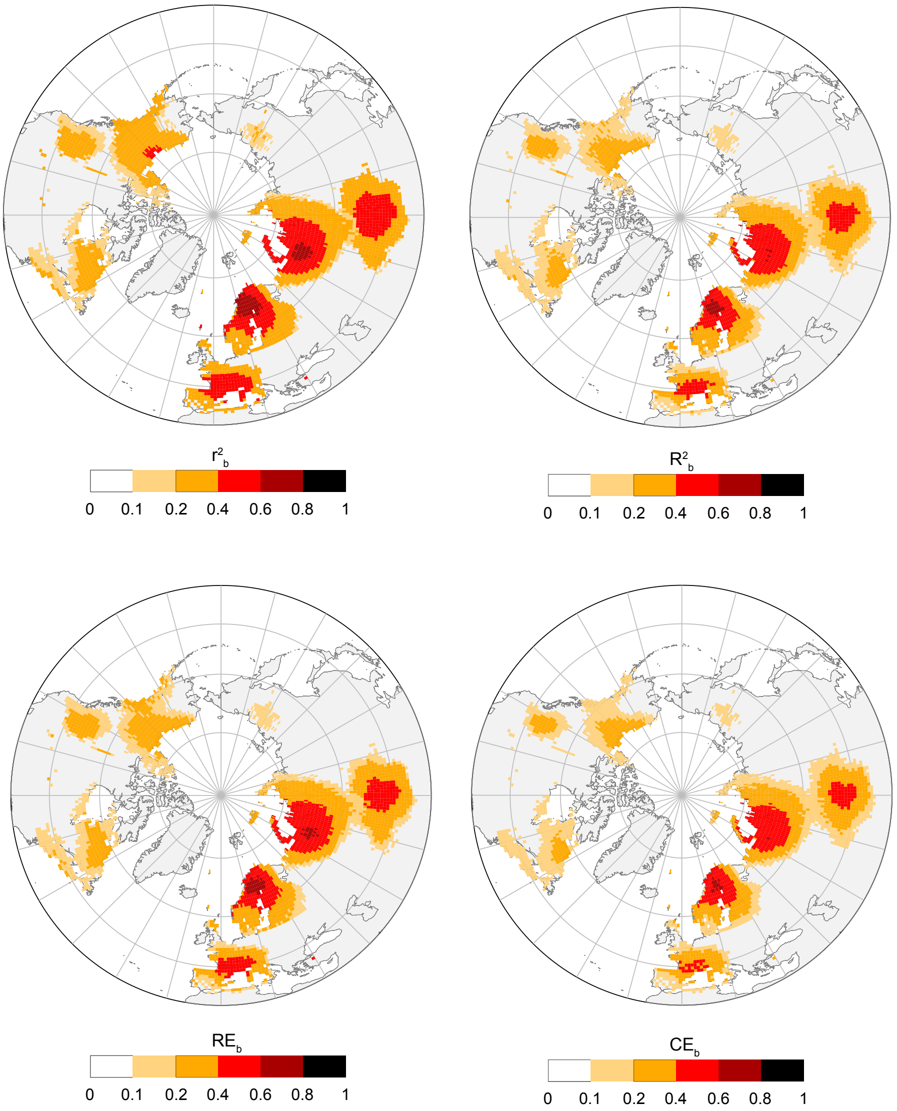

Table S5. Frost rings and light-rings observed in the tree-ring chronologies of the Northern hemisphere in the aftermath of the 1108-1110, 1257 Samalas, 1452 unidentified, 1601 Huaynaputina and 1816 CE Tambora eruptions.

Site	Polar Ural	Yamal	Central Altai	Subarctic Quebec	Western USA
**Time span**	*CE 742-1999*	*CE 742-1999*	CE 500-2007	*CE 706-1676*<br>*CE 1398-1982*	*BCE 3000 - CE 2002*
**Species**	*Juniperus Sibirica*<br>*Larix sibirica* Ledeb.	*Larix sibirica* Ledeb.	*Larix sibirica* Ledeb.	*Picea mariana Mill. BSP*	*Pinus Longaeva*<br>*Pinus Aristata*
**Reference**	*Hantemirov et al. (2004)*	*Hantemirov et al. (2004)*	*Barinov et al. (2017)*	*Arseneault and Payette (2002)*<br>*Fillon et al. (1986)*	*Salzer and Hughes (2007)*
**Type of diagnostic rings**	*Frost rings*	*Frost rings*	*Frost rings*	*Light rings*	*Frost rings*
**1108-1110 eruptions**	*1109*	*1109*	*1109*	*1109*	*1109*
**Samalas eruption**	*1258, 1259*	*1258, 1259*	*1258, 1259, 1260*	*1258*	*1259*
**1452 unidentified eruption**	*1453*	*1453*	-	*1453*	*1453*
**Huaynaputina**	*1601*	*1601*	-	*1601*	*1601*
**Tambora**	-	-	-	*1816*	-

References

Arseneault, D., & Payette, S. (2002). Chronologie des cernes pâles de l'épinette noire (Picea mariana [Mill.] BSP.) au Québec subarctique : de 706 à 1675 ap. J.-C. *Géographie Physique et Quaternaire*, *52*(2), 219–226. https://doi.org/10.7202/004764ar

Barinov, V. V., Myglan, V. S., & Tainik, A. V. (2017). Extreme Climatic Events in the Central Altai of the Last 1500 Years According to Tree-Ring Chronology Jelo. *Izvestiya Rossiiskoi Akademii Nauk. Seriya Geograficheskaya.*, (1), 91–102. https://doi.org/10.15356/0373-2444-2017-1-91-102

Filion, L., Payette, S., Gauthier, L., & Boutin, Y. (1986). Light Rings in Subarctic Conifers as a Dendrochronological Tool. *Quaternary Research*, *26*(2), 272–279. https://doi.org/10.1016/0033-5894(86)90111-0

Hantemirov, R. M., Gorlanova, L. A., & Shiyatov, S. G. (2004). Extreme temperature events in summer in northwest Siberia since AD 742 inferred from tree rings. *Palaeogeography, Palaeoclimatology, Palaeoecology*, *209*(1–4), 155–164. https://doi.org/10.1016/j.palaeo.2003.12.023

Salzer, M. W., & Hughes, M. K. (2007). Bristlecone pine tree rings and volcanic eruptions over the last 5000 yr. *Quaternary Research*, *67*(1), 57–68. https://doi.org/10.1016/j.yqres.2006.07.004

Figure S3. Persistence of climatic anomalies in the Northern hemisphere following the 1108-1110, 1257 Samalas and 1816 CE Tambora eruptions.

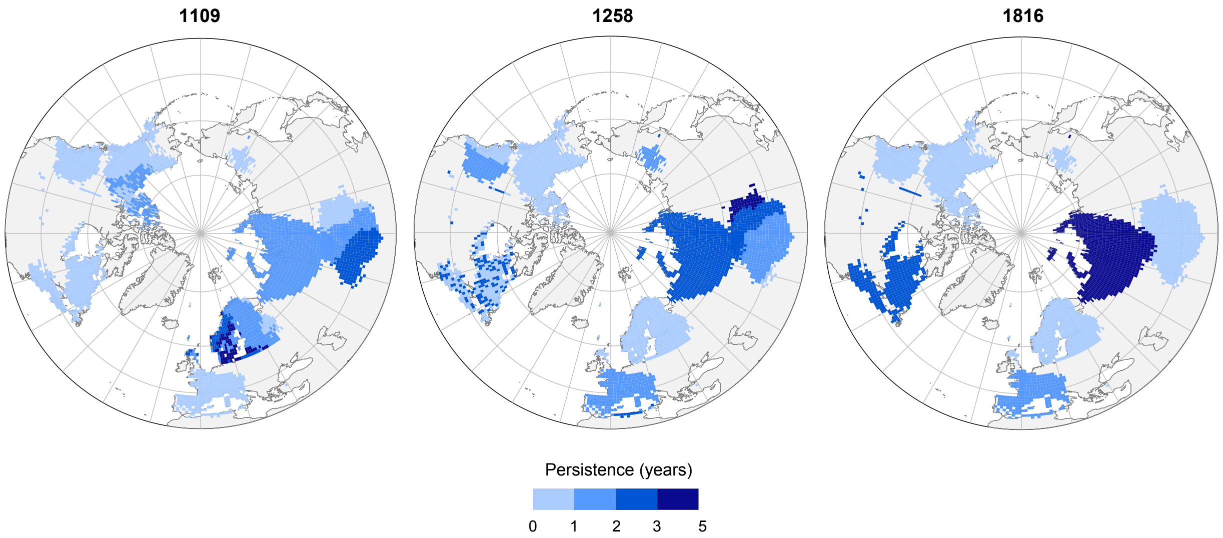

Figure S4. Climatic anomalies and societal impacts reported in European historical archives during the summers of 1109-1110, 1174 and 1195-1196 CE.

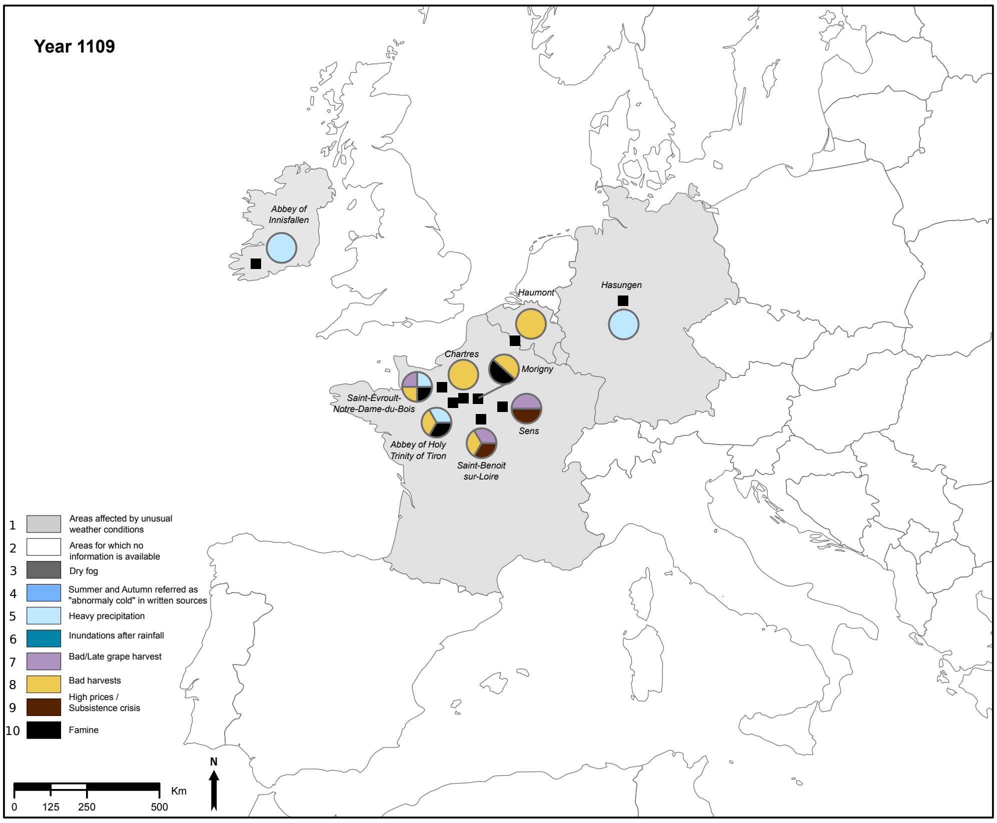

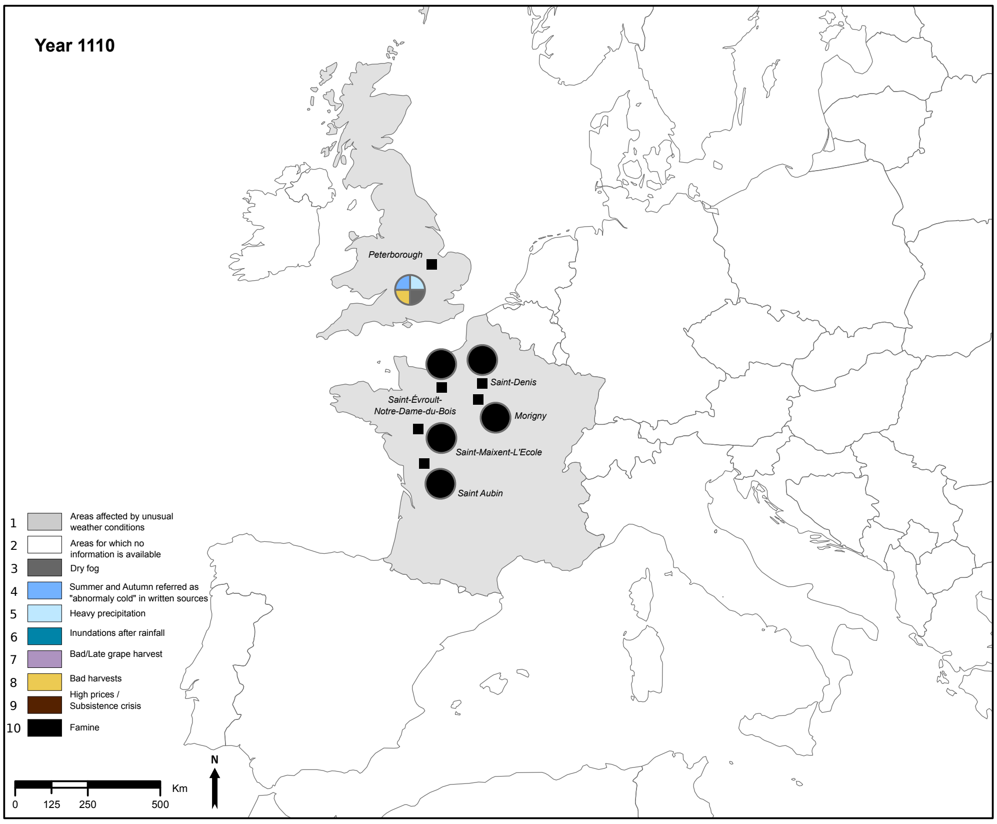

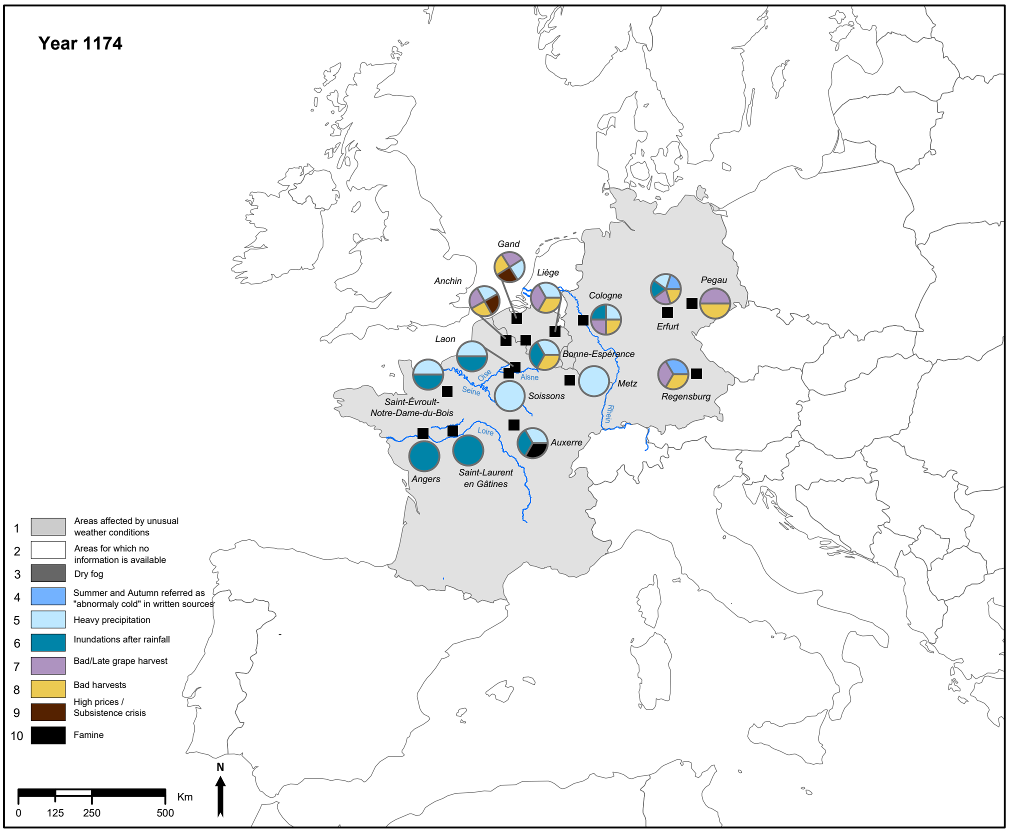

Year 1174
Gand
Anchin
Liège
Cologne
Erfurt
Pegau
Laon
Bonne-Espérance
Saint-Évroult-
Notre-Dame-du-Bois
Soissons
Metz
Regensburg
Auxerre
Angers
Saint-Laurent
en Gâtines
Seine
Oise
Aisne
Loire
Rhein
1 Areas affected by unusual weather conditions
2 Areas for which no information is available
3 Dry fog
4 Summer and Autumn referred as "abnormaly cold" in written sources
5 Heavy precipitation
6 Inundations after rainfall
7 Bad/Late grape harvest
8 Bad harvests
9 High prices / Subsistence crisis
10 Famine
0 125 250 500
Km
N

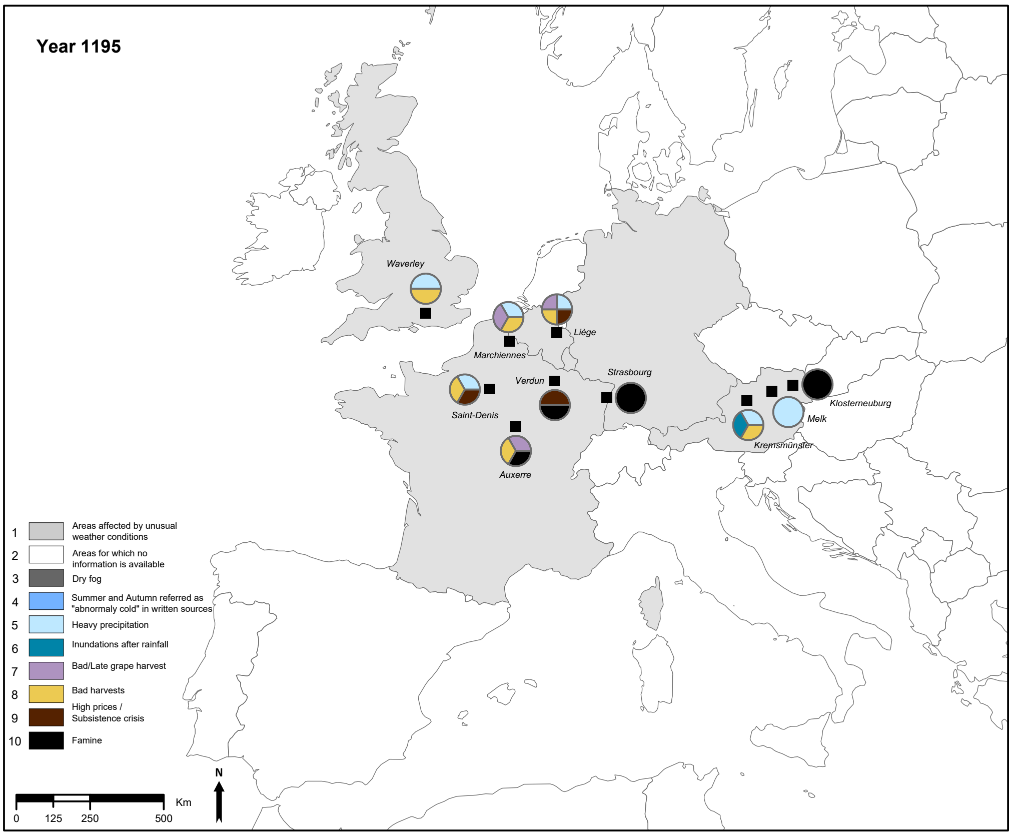
Year 1195
Waverley
Marchiennes
Liége
Verdun
Strasbourg
Saint-Denis
Auxerre
Klosterneuburg
Melk
Kremsmünster
1 Areas affected by unusual weather conditions
2 Areas for which no information is available
3 Dry fog
4 Summer and Autumn referred as "abnormaly cold" in written sources
5 Heavy precipitation
6 Inundations after rainfall
7 Bad/Late grape harvest
8 Bad harvests
9 High prices / Subsistence crisis
10 Famine
N
Km
0 125 250 500

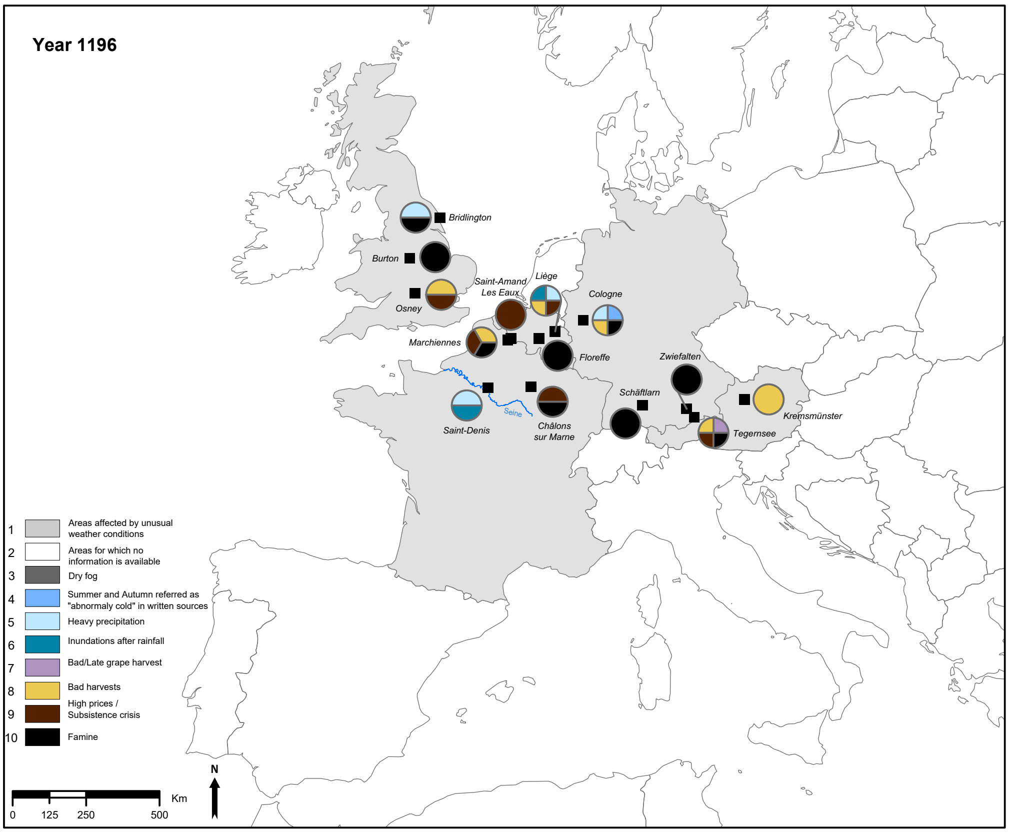
Year 1196
Bridlington
Burton
Osney
Saint-Amand Les Eaux
Liége
Cologne
Marchiennes
Floreffe
Zwiefalten
Schäftlarn
Kremsmünster
Tegernsee
Saint-Denis
Seine
Châlons sur Marne
1 Areas affected by unusual weather conditions
2 Areas for which no information is available
3 Dry fog
4 Summer and Autumn referred as "abnormaly cold" in written sources
5 Heavy precipitation
6 Inundations after rainfall
7 Bad/Late grape harvest
8 Bad harvests
9 High prices / Subsistence crisis
10 Famine
N
Km
0 125 250 500

Figure S5. Number of narrative sources providing weather reports per year and season over the 12th century. Clear peaks are observed following the severe drought of 1137, the cold winter of 1149/1150 and the wet and cold summers of 1109, 1151, 1174, 1195-1196 CE.

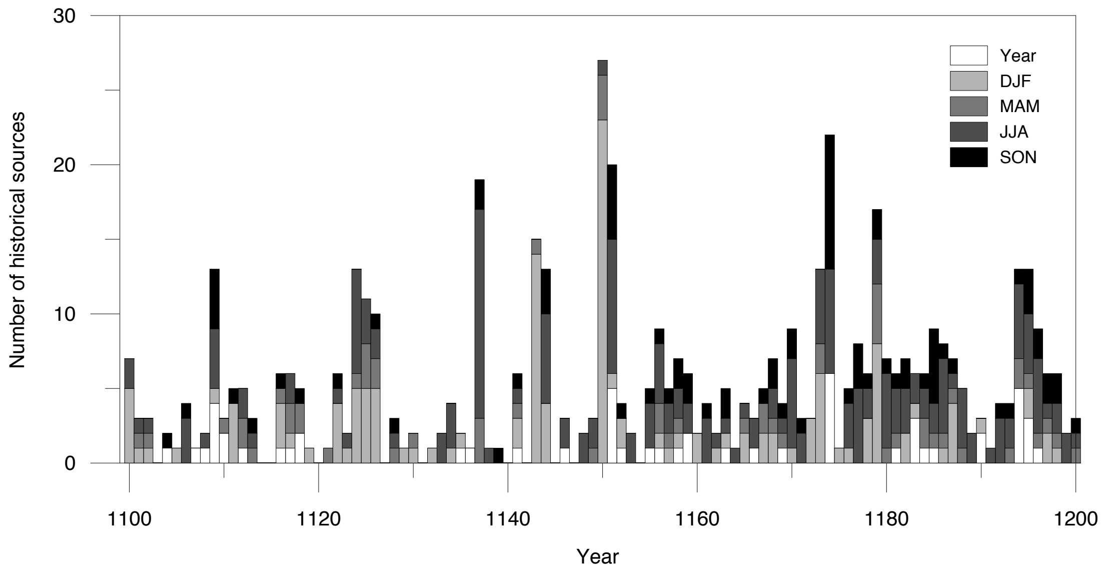

Figure S6. Tree-ring based reconstructions of the warm season Palmer Drought Severity index for Europe for the boreal summer of 1109 CE (Cook et al., 2015), expressed as anomalies from a contemporary reference period (1094–1124 CE).

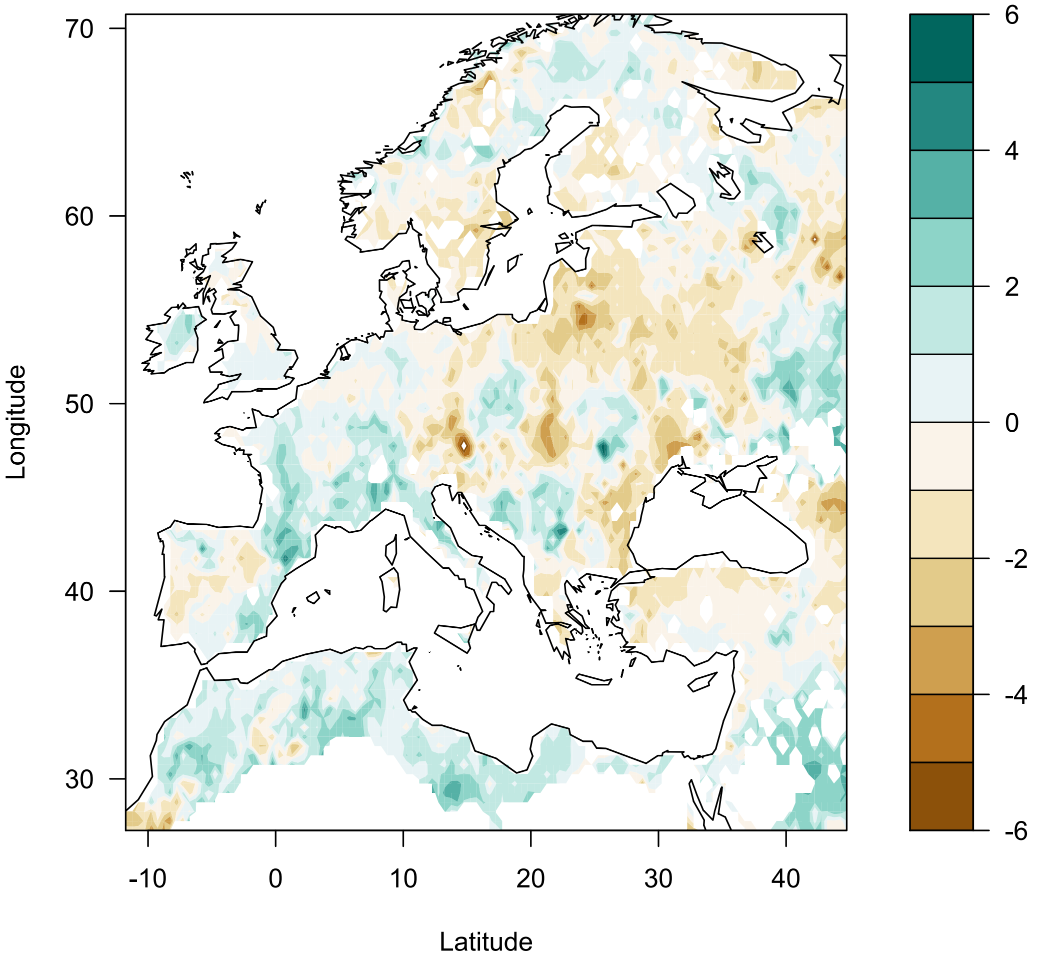

Supplement: Supplementary file 1 — Supplementary Information. [file 41598_2020_63339_MOESM1_ESM.pdf]
